# Impact of self-reported symptoms of allergic rhinitis and asthma on sleep disordered breathing and sleep disturbances in the elderly with polysomnography study

**DOI:** 10.1371/journal.pone.0173075

**Published:** 2017-02-28

**Authors:** Sae-Hoon Kim, Ha-Kyeong Won, Sung-Do Moon, Byung-Keun Kim, Yoon-Seok Chang, Ki-Woong Kim, In-Young Yoon

**Affiliations:** 1 Department of Internal Medicine, Seoul National University College of Medicine, Seoul, South Korea; 2 Institute of Allergy and Clinical Immunology, Seoul National University Medical Research Center, Seoul, South Korea; 3 Department of Internal Medicine, Seoul National University Bundang Hospital, Seongnam, South Korea; 4 Department of Neuropsychiatry, Seoul National University Bundang Hospital, Seongnam, South Korea; St. Michael's Hospital, CANADA

## Abstract

**Background:**

Sleep disordered breathing (SDB) and sleep disturbances have been reported to be associated with allergic rhinitis and asthma. However, population-based studies of this issue in the elderly are rare.

**Objective:**

To investigate the impact of self-reported rhinitis and asthma on sleep apnea and sleep quality using polysomnography in an elderly Korean population.

**Methods:**

A total of 348 elderly subjects who underwent one-night polysomnography study among a randomly selected sample were enrolled. Study subjects underwent anthropometric and clinical evaluations. Simultaneously, the prevalence and co-morbid status of asthma and allergic rhinitis, and subjective sleep quality were evaluated using a self-reported questionnaire.

**Results:**

Ever-diagnosis of allergic rhinitis was significantly more prevalent in subjects with SDB compared with those without SDB. Subjects with an ever-diagnosis of allergic rhinitis showed a higher O_2_ desaturation index and mean apnea duration. Indices regarding sleep efficiency were affected in subjects with a recent treatment of allergic rhinitis or asthma. Waking after sleep onset was longer and sleep efficiency was lower in subjects who had received allergic rhinitis treatment within the past 12 months. Subjects who had received asthma treatment within the past 12 months showed significantly lower sleep efficiency than others.

**Conclusion:**

Our study indicates that a history of allergic rhinitis is associated with increased risk of SDB in the elderly. Sleep disturbance and impaired sleep efficiency were found in the subjects who had received recent treatment of allergic rhinitis or asthma. Physicians should be aware of the high risk of sleep disorders in older patients with respiratory allergic diseases.

## Introduction

In many countries, population ageing is taking place as a result of longer life expectancy and declining fertility rates. Globally, the number of older people (aged 60 years or over) is expected to grow to more than two billion, reaching 21.1% of the world’s population by 2050 [1]. Although sleeping problems are not an inherent part of the ageing process, many older adults suffer from sleeping problems such as sleep apnea and insomnia [2]. Sleep disordered breathing (SDB) is a highly prevalent disorder that commonly occurs in the elderly [3]. The prevalence of insomnia tends to be higher in older individuals with multiple physical and mental conditions [2]. These sleep disorders have a substantial socioeconomic impact on both the individual and society, eliciting negative consequences in quality of life, psychological and physical performance, and mortality [2–4].

In the past, asthma and allergic rhinitis were mostly regarded as diseases of childhood. However, recent literature has indicated the high epidemiologic burden of asthma, and underestimation of allergic rhinitis, in the elderly. Cross sectional surveys have reported that the prevalence of asthma is 4.5%–12.7% in the elderly [5, 6]. Asthma is often more prevalent in the elderly than in younger adults both when examined using a self-administered questionnaire or measurement of airway hyperresponsiveness [7, 8]. Furthermore, recent observations have suggested a high prevalence of rhinitis in the elderly [9, 10]. Results from the National Health and Nutrition Examination Survey found that the self-reported prevalence of rhinitis was approximately 32% in older adults aged between 54 and 89 years, similar to the prevalence in younger adults [9]. Allergic sensitization is generally known to decrease with age [9, 11]. However, recent reports have indicated that allergic rhinitis and local allergic rhinitis are common in the elderly, suggesting that allergic rhinitis may be underestimated in this population [12].

Numerous reports have indicated that SDB and sleep disturbance are commonly associated with asthma and allergic rhinitis [13, 14]. Rhinitis and upper airway disease are well-known risk factors for obstructive sleep apnea (OSA). Nasal obstruction and resistance increases respiratory events and arousals during sleep, leading to sleep fragmentation [14]. OSA and asthma are known to be frequently co-morbid and share risk factors such as obesity, rhinitis, and gastroesophageal reflux [13]. OSA increases the risk for asthma and affects control status, and the risk for OSA is increased in asthmatics [13]. Night-time worsening of asthma is a well-known phenomenon and may induce sleep disturbance [15]. Furthermore, many medications given to treat asthma and allergic rhinitis may affect sleep quality as a result of adverse effects such as drowsiness and insomnia [16]. Despite the large amount of evidence suggesting the impact of asthma and rhinitis on sleep quality, studies conducted in elderly adults using polysomnography assessment of sleep-related disorders are rare. In this study, we investigated the impact of self-reported rhinitis and asthma on sleep apnea and sleep quality using polysomnography in an elderly Korean population.

## Methods

### Study subjects

This study was conducted with cross-sectional manner using a subpopulation of The Korean Longitudinal Study on Cognitive Aging and Dementia, a population-based cohort study in an elderly Korean population aged ≥60 years [17]. We enrolled 348 elderly subjects who agreed to receive a nocturnal polysomnography study among randomly selected samples in Yong-In city, Korea. Sample size was calculated based on assumption of effect size and prevalence of SDB with α error of 0.05 and power (1-β) of 0.85. Prevalence of asthma in SDB group and non-SDB group were expected to be 15% and 5%, respectively based on previous report [18]. Prevalence of SDB in the elderly population was expected to be 30–40%. Study subjects underwent a polysomnography study, and anthropometric and clinical evaluations including height, weight, and neck, waist, and hip circumference, and blood pressure when they received baseline cohort registration work-up. The Pittsburg sleep quality index (PSQI) was used to assess subjective sleep complaints and quality. Simultaneously, prevalence and the comorbid status of asthma and allergic rhinitis were evaluated using questionnaires. This study was approved by institutional review board of Seoul National University Bundang Hospital (approval number: B-1006/103-010) and all subjects provided written informed consent themselves or via their legal guardians.

### Nocturnal polysomnography

One night of polysomnography was recorded using Embla^™^ N7000 (Embla, Reykjavik, Iceland). Briefly, electroencephalography (EEG), electrooculography, and electromyography electrodes were applied at the appropriate positions to record EEG, eye, and muscle movements. Strain gauges were used to record chest and abdominal respiratory movements and nasal pressure cannulas were employed to record airflow. Oxygen saturation was measured using a pulse oximeter applied to the index finger. Sleep was scored at every 30-second epoch of the nocturnal polysomnography based on the criteria of Rechtschaffen and Kales [19]. The apnea and hypopnea index (AHI) was defined as the total number of apneas and hypopneas per hour of sleep. Apnea was defined as complete cessation of airflow for at least 10 seconds. Hypopnea was defined as a clear decrease of airflow (>50%) for at least 10 seconds or a discernible reduction in airflow for at least 10 seconds associated with electroencephalographic arousal or oxygen desaturation [20]. SDB usually stands for sleep apnea syndrome, although it is broad term describing disorders of respiratory pattern or quantity of ventilation that occur periodically during sleep. SBD was defined as obstructive and central sleep apnea, and the criterion for SDB was defined as AHI≥15 in this study [21]. In-addition, sleep-related parameters including waking after sleep onset, sleep onset latency, sleep efficiency (the number of minutes of sleep divided by the number of minutes in bed), respiratory arousal, lowest O_2_ saturation, O_2_ desaturation index, and mean apnea duration were analyzed to assess sleep quality and sleep-disordered breathing status.

### Questionnaire for co-morbidity of asthma and rhinitis

Evaluations for the prevalence and co-morbidity of asthma and allergic rhinitis were performed using a self-administered questionnaire, which was modified from the Korean adult general population asthma & rhinitis survey questionnaire [22, 23]. The questionnaire consisted of nine main questions and seven collateral questions asking about symptoms and a medical history of asthma and allergic rhinitis. Regarding asthma, subjects were asked the following: (i) Have you experienced any wheezing during the past 12 months? (ii) Have you ever been diagnosed with asthma? (iii) Have you received any treatment for asthma during the past 12 months? Regarding allergic rhinitis, subjects were asked the following: (i) Have you ever experienced sneezing and a stuffy nose during the past 12 months when you did not have a cold? (ii) Have you ever been diagnosed with allergic rhinitis? (iii) Have you received any treatment for allergic rhinitis during the past 12 months? Current asthma was defined as when the subject had current wheezing and a physician-made ever-diagnosis of asthma together.

### Statistical analysis

The t-test for continuous variables and chi-square test or Fisher exact test (for nonparametric results) for categorical variables was used to compare the clinical characteristics and prevalence of asthma and allergic rhinitis according to the presence of SDB. Univariate and multivariate analysis of affecting factors for SDB were performed with age, gender, BMI, neck circumference and history of allergic rhinitis using logistic regression test. The Mann-Whitney test was used to compare nonparametric sleep-related variables according to the co-morbid conditions of asthma and allergic rhinitis. Statistical analyses were performed using SPSS ver. 18.0 (SPSS, Inc., Chicago, IL, USA); *p*<0.05 was deemed to indicate statistical significance.

## Results

### Ever-diagnosis of allergic rhinitis as a risk factor for sleep-disordered breathing

A total of 127 subjects (36.5%) were diagnosed with SDB among the 348 participants when it was defined as AHI ≥15. SDB was significantly more predominant among male subjects. Subjects with SDB had higher BMI, and waist, hip, and neck circumference when compared with subjects without SDB. Diastolic BP was higher in the SDB group than the subjects without SDB, although there was no significant difference in systolic BP. Subjects with SDB showed higher rates of respiratory arousal, higher O_2_ desaturation index, longer mean apnea duration, and lower lowest O_2_ compared with subjects without SDB. However, high SDB was not related to sleep efficiency, waking after sleep onset, or PSQI. (Table 1)

**Table 1:** Demographic and sleep characteristics of study subjects.

Characteristics	Total (N = 348)	AHI<15 (N = 221)	AHI≥15 (N = 127)	p-value
Age	68.3±5.6	67.9±5.6	68.9±5.6	0.151
Gender(male)	135 (38.8%)	64 (29.0%)	71 (55.9%)	<0.001
BMI	23.7±2.7	23.2±2.5	24.5±3.0	<0.001
Waist circumference	84.8±9.1	83.3±9.3	87.4±8.2	<0.001
Hip circumference	94.5±5.7	93.5±5.2	96.3±6.1	<0.001
Neck circumference	37.4±4.0	36.6±3.4	38.8±4.5	<0.001
SBP	114.8±15.2	114.3±16.0	115.9±13.7	0.340
DBP	74.3±9.1	73.4±9.2	75.8±9.0	0.019
Waking after sleep onset	95.9±62.0	93.1±58.6	100.7±67.6	0.273
Total sleep time	374.3±67.0	375.9±64.9	371.6±70.9	0.572
Sleep onset latency	18.8±28.2	18.3±24.8	19.9±33.6	0.621
Sleep efficiency	76.9±13.4	77.3±13.0	76.1±13.9	0.418
Respiratory arousal	10.4±11.9	3.6±2.9	22.1±12.4	<0.001
Lowest O2	86.0±5.6	88.0±4.2	82.7±6.0	<0.001
O2 desaturation index	10.9±12.8	4.2±7.1	22.6±12.3	<0.001
Mean apnea duration	18.2±9.2	15.6±9.4	22.8±6.8	<0.001
Pittsburg Sleep Quality Index	6.5±3.7	6.7±3.8	6.2±3.6	0.210

BMI, body mass index; SBP, systolic blood pressure; DBP, diastolic blood pressure

We analyzed the self-reported prevalence of asthma and allergic rhinitis among subjects with SDB. Self-reported ever-diagnosis of allergic rhinitis was significantly more prevalent in the subjects with SDB compared with those without SDB (22.6% vs. 13.6%, adjusted p = 0.046). No significant difference between groups was observed in the presence of current asthma, ever-diagnosis of asthma, asthma treatment within the past 12 months, rhinitis symptoms within the past 12 months, or allergic rhinitis treatment within the past 12 months. (Table 2) Multivariate analysis revealed history of allergic rhinitis (self-reported ever-diagnosis of allergic rhinitis) is a significant risk factors affecting SDB after adjustment of other risk factors of SDB including age, gender, BMI, and neck circumference. (Table 3)

**Table 2:** Prevalence of asthma and rhinitis according to sleep disordered breathing.

Clinical features	Total (N = 348)	AHI<15 (N = 221)	AHI≥15 (N = 127)	p-value
Current asthma	9 (2.6%)	8 (3.7%)	1 (0.8%)	0.163
Ever-diagnosis of asthma	21 (6.2%)	14 (6.5%)	7 (5.6%)	0.750
Asthma treatment, past 12 months	15 (4.4%)	9 (4.1%)	6 (4.6%)	0.777
Rhinitis symptoms, past 12 months	96 (28.7%)	58 (27.6%)	38 (30.4%)	0.586
Ever-diagnosis of allergic rhinitis	57 (16.9%)	29 (13.6%)	28 (22.6%)	0.034
Allergic rhinitis, past 12 months	32 (9.4%)	16 (7.5%)	16 (12.7%)	0.114

**Table 3:** Univariate and multivariate analysis of affecting factors for sleep disordered breathing.

Variables	Unadjusted		Adjusted*	
	OR (95% CI)	p-value	OR (95% CI)	p-value
Age	1.03 (0.99–1.07)	0.151	1.03 (0.99–1.08)	0.127
Gender	3.11 (1.97–4.90)	<0.001	2.99 (1.65–5.41)	<0.001
BMI	1.21 (1.11–1.32)	<0.001	1.23 (1.11–1.36)	<0.001
Neck circumference	1.19 (1.11–1.28)	<0.001	1.02 (0.97–1.11)	0.654
History of allergic rhinitis	1.85 (1.04–3.29)	0.036	1.90 (1.01–3.57)	0.046

*adjusted for all variables shown using logistic regression test

### Impairment of sleep quality in subjects with recent treatment of asthma or allergic rhinitis

Sleep quality and SDB-related parameters were affected by comorbid allergic rhinitis more than comorbid asthma. Subjects with self-reported ever-diagnosis of allergic rhinitis showed a higher O_2_ desaturation index (13.9±15.2 vs. 10.5±12.4, p = 0.022) and mean apnea duration (21.9±10.8 vs. 17.6±8.7, p = 0.027), although AHI was not significantly different. On the other hand, indices regarding sleep disturbances were affected in subjects who had received allergic rhinitis treatment within the past 12 months. Waking after sleep onset was longer (123.1±71.4 vs. 93.9±60.8, p = 0.017) and sleep efficiency was lower (71.7±14.6 vs. 77.3±13.2, p = 0.028) in subjects who had received allergic rhinitis treatment within the past 12 months. The PSQI score was also significantly higher in the allergic rhinitis treatment within the past 12 months group (8.2±4.5 vs. 6.4±3.6, p = 0.024). Subjects with rhinitis symptoms within the past 12 months showed a higher PSQI (7.5±3.9 vs. 6.1±3.5, p = 0.001) and longer mean apnea duration (20.5±9.4 vs. 17.6±9.0, p = 0.037). (Table 4) In addition, the proportions of sleep stages were analyzed according to the comorbid status of rhinitis and asthma. The percentage of non-rapid eye movement (non-REM) stage 2 sleep was significantly lower in subjects with an ever-diagnosis of allergic rhinitis than in those without a diagnosis (42.3±9.9 vs. 46.7±11.6, p = 0.036). Subjects who had rhinitis symptoms within the past 12 months showed significantly lower percentage of non-REM stage 3 sleep indicating deep sleep (5.7±6.1 vs. 6.9±5.3, p = 0.017). In the analysis of sleep parameters according to comorbid asthma, subjects who had received asthma treatment within the past 12 months showed significantly lower sleep efficiency than others (68.9±16.7 vs. 77.2±13.2, p = 0.043). Otherwise, we could not find any associations between sleep parameters and asthma in our study subjects. (Table 5)

**Table 4:** Sleep parameters in polysomnography and sleep quality index according to co-morbidity of allergic rhinitis.

Parameters	Rhinitis symptoms, past 12 months		p-value	Ever-diagnosis of allergic rhinitis		p-value	Allergic rhinitis treatment, past 12 months		p-value
	Yes (N = 96)	No (N = 239)		Yes (N = 57)	No (N = 280)		Yes (N = 32)	No (N = 307)	
Apnea Hypopnea Index	16.1±14.5	15.0±14.8	0.283	16.7±13.7	15.0±15.0	0.112	17.0±15.6	15.1±14.7	0.443
Obstructive apnea index	5.9±9.0	5.8±9.5	0.256	6.4±8.7	5.7±9.4	0.055	6.9±9.9	5.7±9.2	0.216
Central apnea index	0.6±1.2	0.6±2.2	0.591	0.6±1.3	0.6±2.1	0.834	0.5±0.9	0.6±2.1	0.686
Mixed apnea index	0.8±2.8	0.6±2.9	0.992	0.9±2.4	0.6±2.9	0.090	0.9±2.2	0.6±2.9	0.072
Hypopnea index	8.8±7.4	8.0±7.0	0.230	8.8±6.8	8.1±7.4	0.205	8.8±7.7	8.1±7.3	0.645
Waking after sleep onset	105.3±71.3	93.4±58.6	0.310	106.1±68.6	94.5±59.0	0.274	123.1±71.4	93.9±60.8	0.017
Sleep efficiency	75.4±14.7	77.3±13.0	0.424	75.0±14.6	77.0±13.1	0.369	71.7±14.6	77.3±13.2	0.028
Stage 1 sleep (%)	13.8±6.9	14.0±7.5	0.890	13.4±6.5	14.0±7.4	0.732	13.7±7.4	13.9±7.3	0.836
Stage 2 sleep (%)	45.1±11.2	46.2±11.6	0.242	44.5±10.8	46.3±11.7	0.352	42.3±9.9	46.5±11.7	0.036
Stage 3 sleep (%)	5.7±6.1	6.9±5.3	0.017	5.6±4.9	6.7±5.6	0.165	5.5±5.0	6.6±5.7	0.310
REM sleep (%)	15.3±6.0	14.8±5.8	0.550	15.7±5.6	14.8±5.9	0.286	14.4±5.7	15.0±5.8	0.661
Respiratory arousal	11.1±11.6	10.3±12.2	0.243	11.3±11.4	10.3±12.1	0.211	11.9±12.6	10.4±11.9	0.399
Lowest O2	85.2±6.2	86.2±5.3	0.131	85.1±5.0	86.2±5.6	0.064	85.7±5.5	86.0±5.6	0.712
O2 desaturation index	12.3±14.6	10.6±12.2	0.296	13.9±15.2	10.5±12.4	0.022	14.8±18.6	10.7±12.2	0.237
Mean apnea duration	20.5±9.4	17.6±9.0	0.037	21.9±10.8	17.6±8.7	0.027	22.4±11.7	17.9±8.8	0.088
Pittsburg Sleep Quality Index	7.5±3.9	6.1±3.5	0.001	7.5±4.3	6.4±3.6	0.087	8.2±4.5	6.4±3.6	0.024

REM sleep, rapid eye movement sleep

**Table 5:** Sleep parameters in polysomnography and sleep quality index according to co-morbidity of asthma.

Parameters	Current asthma		p-value	Ever-diagnosis of asthma		p-value	Asthma treatment, past 12 months		p-value
	Yes (N = 9)	No (N = 337)		Yes (N = 21)	No (N = 318)		Yes (N = 15)	No (N = 327)	
Apnea Hypopnea Index	7.2±6.6	15.3±14.8	0.091	16.6±21.1	15.1±14.3	0.569	18.7±22.9	15.0±14.2	0.935
Obstructive apnea index	0.3±1.2	5.8±9.3	0.173	8.9±17.2	5.6±8.5	0.819	11.1±20.0	5.5±8.4	0.709
Central apnea index	0.2±0.4	0.6±2.0	0.711	0.2±0.3	0.6±2.0	0.455	0.2±0.4	0.6±2.0	0.926
Mixed apnea index	0.0±0.1	0.7±2.9	0.187	0.0±0.1	0.7±2.9	0.354	0.1±0.1	0.7±2.9	0.550
Hypopnea index	5.7±5.9	8.2±7.3	0.239	7.4±8.5	8.3±7.2	0.278	7.3±6.7	8.2±7.3	0.677
Waking after sleep onset	96.2±54.8	96.1±62.4	0.768	117.3±72.5	94.5±59.9	0.152	127.5±72.6	95.0±61.5	0.062
Sleep efficiency	72.8±17.1	76.9±13.3	0.451	70.3±17.6	77.2±13.1	0.082	68.9±16.7	77.2±13.2	0.043
Stage 1 sleep (%)	9.9±4.4	13.9±7.3	0.076	13.9±7.4	13.9±7.3	0.942	13.6±7.7	13.9±7.3	0.801
Stage 2 sleep (%)	45.3±11.1	46.2±11.7	0.855	42.6±12.3	46.2±11.5	0.206	41.0±12.2	46.3±11.6	0.107
Stage 3–4 sleep (%)	9.0±6.1	6.5±5.6	0.185	6.4±6.2	6.5±5.5	0.686	6.8±6.2	6.5±5.6	0.990
REM sleep (%)	17.2±7.7	14.9±5.6	0.403	14.1±7.7	15.0±5.7	0.423	13.8±8.1	15.0±5.7	0.388
Respiratory arousal	3.7±3.3	10.5±12.0	0.073	10.9±17.1	10.4±11.6	0.374	13.4±19.6	10.3±11.5	0.928
Lowest O2	87.6±2.7	86.0±5.6	0.493	87.4±3.2	85.9±5.7	0.411	86.8±3.0	86.0±5.6	0.892
O2 desaturation index	5.0±5.5	11.1±13.0	0.174	11.6±14.2	11.0±12.9	0.810	12.8±14.1	10.8±12.9	0.591
Mean apnea duration	16.0±4.2	18.3±9.4	0.175	18.7±4.9	18.3±9.4	0.908	18.5±5.7	18.3±9.4	0.817
Pittsburg Sleep Quality Index	5.8±2.2	6.6±3.8	0.824	6.8±3.5	6.5±3.7	0.593	6.2±3.0	6.5±3.8	0.975

REM sleep, rapid eye movement sleep

## Discussion

In the present study, we performed nocturnal polysomnography in an elderly Korean population randomly selected from the community. The prevalence of SDB, defined as AHI≥15, was as high as 36.5% and subjects with SDB showed higher respiratory arousal, O_2_ desaturation index, and mean apnea duration. SDB is an umbrella term used to describe disorders of respiratory pattern or quantity of ventilation that occur periodically during sleep, and is commonly associated with OSA [3]. OSA is characterized by partial or complete cessation of airflow and oxygen desaturation during sleep as a result of upper airway collapse. The nose accounts for a large part of the total resistance of the upper airway and any factors causing nasal obstruction can lead to snoring or apnea [14]. Elevation of nasal resistance can also promote inspiratory collapse at the pharyngeal level increasing negative pressure in the upper airway. Chronic nasal inflammatory conditions, such as allergic rhinitis, appear to contribute to upper airway obstruction in OSA [24]. Numerous studies have demonstrated that allergic rhinitis can predispose an individual to or worsen the symptoms of OSA. It was reported that subjects with persistent nasal congestion owing to allergy are 1.8 times more likely to have moderate to severe SDB than subjects without nasal congestion from allergy [25]. In accordance with previous reports, our study showed that SDB was associated with male sex, high BMI, large neck circumference, and high blood pressure. Furthermore, a self-reported history (ever-diagnosis by physicians) of allergic rhinitis was significantly more prevalent in subjects with SDB even after adjusting for confounding factors including age, gender, BMI, and neck circumference. This data indicates that a history of allergic rhinitis is associated with increased risk of SDB in the elderly population.

Allergic rhinitis has been recognized relatively less commonly in the elderly. It has been long believed that allergic sensitization diminishes with age and non-allergic rhinitis is more common in the elderly. The low incidence and high remission rate of allergic sensitization were reported in the long-term follow up of an adult population [11]. However, rhinitis is also prevalent in the elderly population with prevalence as high as 32%, which was not significantly different from that in younger adults [9]. Some reports have indicated that the change in rhinitis symptoms seems not related with the change of skin test reactivity in older adults [26, 27]. Non-allergic rhinitis at the initial evaluation can often be converted into allergic rhinitis at the second evaluation in the adult population [28]. Moreover, recent studies have revealed a subtype of allergic rhinitis which just has localized IgE-mediated allergic inflammation in the nasal mucosa without a positive skin test or blood-specific IgE to aeroallergen [29]. A high prevalence of local allergic rhinitis has been observed in elderly patients with rhinitis [12]. These data implicate that a substantial proportion of elderly patients with rhinitis might have an aeroallergen allergy and persistence of symptoms despite their low skin test reactivity.

In our study, only self-reported ever-diagnosis of allergic rhinitis was associated with SDB, while rhinitis symptoms or treatment of allergic rhinitis within the past 12 months were not. This result might imply that a clinical history of nasal allergy itself is a more critical risk factor for SDB rather than nasal symptoms such as obstruction or discharge, although the exact mechanism is not clear. Some reports have highlighted OSA as a pro-inflammatory disorder that co-aggregates with obesity, interacting with respiratory allergic diseases [30]. Chronic local and systemic inflammatory conditions in subjects who have an ever-diagnosis of allergic rhinitis may affect the development and severity of SDB. Interestingly, ever-diagnosis of allergic rhinitis had a negative influence on parameters related with SDB such as the O_2_ desaturation index and mean apnea duration, but did not significantly affect sleep efficiency as measured using polysomnography and PSQI. Meanwhile, treatment for allergic rhinitis or rhinitis symptoms within the past 12 months contribute to poor sleep quality and sleep efficiency more than parameters related with SDB. Insomnia and SDB could be co-occurring [31], but some studies have revealed inconsistent results about their co-occurrence [32, 33]. The degree of SDB does not seem to be always correlated with sleep efficiency or subjective sleep quality. Characteristics of sleep disturbance, sleep apnea, or insomnia might differ according to the comorbid conditions of rhinitis such as the type of rhinitis, severity of symptoms and inflammation, disease duration, upper airway complications, and medications. Nasal obstruction or airflow resistance in the upper airway can contribute to both poor sleep efficiency and sleep apnea. However, nasal discharge and an itching sensation is likely to impair sleep efficiency eliciting insomnia and waking rather than SDB [34]. Many medications used in the treatment of allergic rhinitis may have potential adverse effects on patients’ sleep quality. Although first generation antihistamine can be used to treat insomnia because of their sedative effect, decongestants frequently combined with antihistamines are well-known to cause insomnia [35]. A review of the pharmacovigilance program of the World Health Organization revealed that intranasal corticosteroids as well as systemic corticosteroids can be complicated by neuropsychiatric adverse reactions including insomnia [36]. Older adults are more prone to sleep disturbance than younger adults [2]. Our data support that physicians need to pay more attention to poor sleep quality and efficiency in elderly patients who have current symptoms of rhinitis and are taking rhinitis medications.

Many epidemiologic studies reported a high risk of OSA in asthmatic patients and a high risk of asthma in adults with OSA [13]. These two diseases are bi-directionally linked with overlapping risk factors such as rhinitis, obesity, and gastroesophageal reflux [13, 30]. OSA may have an influence on asthmatic symptom control and asthma-related quality of life [37, 38]. Suggested potential pathophysiologic mechanisms include neuromechanical effects of upper airway collapse, systemic and airway inflammation enhanced by chronic intermittent hypoxia or obesity, and sleep fragmentation [13, 30]. In addition, several studies have suggested that taking oral or inhaled corticosteroids may lead to the development of OSA through parapharyngeal fat deposition and myopathy because of corticosteroid use [39, 40]. In contrast to the large amount of evidence in patients with asthma or OSA, population-based association studies are rare. One European study in the general population reported a high prevalence of snoring (17%) and witnessed apnea (14%) in subjects with physician-diagnosed asthma compared with the overall prevalence [41]. A recent population-based prospective study demonstrated asthma is associated with increased risk of new onset OSA [42]. However, population-based studies utilizing polysomnography to identify the prevalence and severity of OSA in asthma have not been reported yet, especially in elderly populations. We evaluated the associations between SDB and asthma in an elderly Korean population using polysomnography, but an association was not found in our study. Although the small number of current asthmatics and subjects with a self-reported ever-diagnosis of asthma might have limited adequate evaluation, our data suggest a possibility that the linkage between OSA and asthma is not clear in the elderly. Elderly asthma is reported to be considerably distinct from the disease in childhood and young adults in terms of clinical characteristics and risk factors [43]. Further studies are warranted to clarify this issue more clearly in the future. Meanwhile, it is noticeable that subjects with asthma treatment within the past 12 months showed significantly poorer sleep efficiency on polysomnography, similar to the case for allergic rhinitis. This suggests that other types of sleep disturbance may happen in asthmatics who have symptoms or have taken asthma medications recently.

Our study has several limitations. First, the prevalence of asthma and allergic rhinitis were evaluated using a self-reported questionnaire without objective tests such as spirometry with bronchodilator response, a bronchial provocation test, or allergy skin test. Recall bias can be an issue in the elderly population. Although the questions about diagnosis of asthma and allergic rhinitis were based on previous diagnosis by a physician, it is possible that other differential medical conditions, such as chronic obstructive pulmonary disease or non-allergic rhinitis, might be misdiagnosed in the elderly. However, chronic obstructive pulmonary disease would not be a big problem in our study subjects. Smoking did not affect prevalence of current asthma and recent asthma treatment at all. (data not shown) There was no current smoker in subjects with ever-diagnosed asthma or recent asthma treatment. Proportion of ex-smoker who had smoked over 10pack year was also small. (4 of 21 subject with ever-diagnosed asthma and 2 of 15 subjects with recent asthma treatment) Second, the sample size of the study was insufficient to evaluate the association between asthma and OSA as mentioned earlier. Originally the sample size of the study was determined based on the expected prevalence of SDB in the elderly. Some previous studies have reported a prevalence of rhinitis and asthma as high as 32% and 12.7%, respectively. However, the self-reported prevalence of asthma and rhinitis was lower than our expectation in this study. In particular, the small population of asthmatics in our study subjects limited the adequate evaluation of the association between asthma and OSA.

Nonetheless, our study has important clinical implications in managing elderly patients who have sleep disorders or respiratory allergic diseases. We demonstrated that a history of allergic rhinitis can be a risk factor for SDB in the elderly as well as younger adults. In the polysomnography study of an elderly population, sleep disturbance and impaired sleep efficiency were found in the subjects who had received recent treatment of allergic rhinitis or asthma. Physicians should be aware that older patients with allergic rhinitis and asthma are susceptible to sleep problems when they have active symptoms or take medications for these diseases.

## Supporting information

S1 Table Dataset of key variables of the study subjects. (DOCX)

## References

[1] United Nations DoEaSA, Population Division. World Population Aeging 2013. ST/SEA/SERA/348. 2013.

[2] Rodriguez JC, Dzierzewski JM, Alessi CA. Sleep problems in the elderly. The Medical clinics of North America. 2015;99(2):431–9. 10.1016/j.mcna.2014.11.013 25700593 PMC4406253

[3] McMillan A, Morrell MJ. Sleep disordered breathing at the extremes of age: the elderly. Breathe. 2016;12(1):50–60. 10.1183/20734735.003216 27064674 PMC4818236

[4] Jennum P, Kjellberg J. Health, social and economical consequences of sleep-disordered breathing: a controlled national study. Thorax. 2011;66(7):560–6. 10.1136/thx.2010.143958 21199816

[5] Song WJ, Cho SH. Challenges in the Management of Asthma in the Elderly. Allergy, asthma & immunology research. 2015;7(5):431–9. 10.4168/aair.2015.7.5.431 PMC4509655 26122503

[6] Baptist AP, Nyenhuis S. Rhinitis in the Elderly. Immunology and allergy clinics of North America. 2016;36(2):343–57. 10.1016/j.iac.2015.12.010 27083107 PMC4834138

[7] Bauer BA, Reed CE, Yunginger JW, Wollan PC, Silverstein MD. Incidence and outcomes of asthma in the elderly. A population-based study in Rochester, Minnesota. Chest. 1997;111(2):303–10. 9041973 10.1378/chest.111.2.303

[8] Song WJ, Kang MG, Chang YS, Cho SH. Epidemiology of adult asthma in Asia: toward a better understanding. Asia Pacific allergy. 2014;4(2):75–85. 10.5415/apallergy.2014.4.2.75 24809012 PMC4005350

[9] Shargorodsky J, Garcia-Esquinas E, Galan I, Navas-Acien A, Lin SY. Allergic Sensitization, Rhinitis and Tobacco Smoke Exposure in US Adults. PloS one. 2015;10(7):e0131957 10.1371/journal.pone.0131957 26172447 PMC4501790

[10] Slavin RG. Special considerations in treatment of allergic rhinitis in the elderly: role of intranasal corticosteroids. Allergy and asthma proceedings: the official journal of regional and state allergy societies. 2010;31(3):179–84. 10.2500/aap.2010.31.3342 20615319

[11] Warm K, Backman H, Lindberg A, Lundback B, Ronmark E. Low incidence and high remission of allergic sensitization among adults. The Journal of allergy and clinical immunology. 2012;129(1):136–42. 10.1016/j.jaci.2011.08.033 21975174

[12] Bozek A, Ignasiak B, Kasperska-Zajac A, Scierski W, Grzanka A, Jarzab J. Local allergic rhinitis in elderly patients. Annals of allergy, asthma & immunology: official publication of the American College of Allergy, Asthma, & Immunology. 2015;114(3):199–202. 10.1016/j.anai.2014.12.013 25744906

[13] Prasad B, Nyenhuis SM, Weaver TE. Obstructive sleep apnea and asthma: associations and treatment implications. Sleep medicine reviews. 2014;18(2):165–71. 10.1016/j.smrv.2013.04.004 23890469

[14] Wilhelm CP, deShazo RD, Tamanna S, Ullah MI, Skipworth LB. The nose, upper airway, and obstructive sleep apnea. Annals of allergy, asthma & immunology: official publication of the American College of Allergy, Asthma, & Immunology. 2015;115(2):96–102. 10.1016/j.anai.2015.06.011 26250769

[15] Fitzpatrick MF, Martin K, Fossey E, Shapiro CM, Elton RA, Douglas NJ. Snoring, asthma and sleep disturbance in Britain: a community-based survey. The European respiratory journal. 1993;6(4):531–5. 8491303

[16] Novak M, Shapiro CM. Drug-induced sleep disturbances. Focus on nonpsychotropic medications. Drug safety. 1997;16(2):133–49. 9067124 10.2165/00002018-199716020-00005

[17] Lee SD, Kang SH, Ju G, Han JW, Kim TH, Lee CS, et al The prevalence of and risk factors for sleep-disordered breathing in an elderly Korean population. Respiration; international review of thoracic diseases. 2014;87(5):372–8. 10.1159/000358442 24714628

[18] Bhattacharyya N, Kepnes LJ. Ambulatory office visits and medical comorbidities associated with obstructive sleep apnea. Otolaryngology—head and neck surgery: official journal of American Academy of Otolaryngology-Head and Neck Surgery. 2012;147(6):1154–7. 22951431 10.1177/0194599812459850

[19] Rechtschaffen A K A. A manual of standardized terminology, technique, and scoring system for sleep stages of human subjects. Los Angeles, University of California Brain Information Service/Brain Research Institute 1968.

[20] Sleep-related breathing disorders in adults: recommendations for syndrome definition and measurement techniques in clinical research. The Report of an American Academy of Sleep Medicine Task Force. Sleep. 1999;22(5):667–89. 10450601

[21] Nieto FJ, Young TB, Lind BK, Shahar E, Samet JM, Redline S, et al Association of sleep-disordered breathing, sleep apnea, and hypertension in a large community-based study. Sleep Heart Health Study. Jama. 2000;283(14):1829–36. 10770144 10.1001/jama.283.14.1829

[22] Rhee CS, Wee JH, Ahn JC, Lee WH, Tan KL, Ahn S, et al Prevalence, risk factors and comorbidities of allergic rhinitis in South Korea: The Fifth Korea National Health and Nutrition Examination Survey. American journal of rhinology & allergy. 2014;28(2):e107–14. 24717946 10.2500/ajra.2014.28.4040

[23] Cheng HM, Kim S, Park GH, Chang SE, Bang S, Won CH, et al Low vitamin D levels are associated with atopic dermatitis, but not allergic rhinitis, asthma, or IgE sensitization, in the adult Korean population. The Journal of allergy and clinical immunology. 2014;133(4):1048–55. 10.1016/j.jaci.2013.10.055 24388009

[24] Staevska MT, Mandajieva MA, Dimitrov VD. Rhinitis and sleep apnea. Current allergy and asthma reports. 2004;4(3):193–9. 15056401 10.1007/s11882-004-0026-0

[25] Young T, Finn L, Kim H. Nasal obstruction as a risk factor for sleep-disordered breathing. The University of Wisconsin Sleep and Respiratory Research Group. The Journal of allergy and clinical immunology. 1997;99(2):S757–62. 9042068 10.1016/s0091-6749(97)70124-6

[26] Simola M, Holopainene E, Malmberg H. Changes in skin and nasal sensitivity to allergens and the course of rhinitis; a long-term follow-up study. Annals of allergy, asthma & immunology: official publication of the American College of Allergy, Asthma, & Immunology. 1999;82(2):152–6. 10.1016/S1081-1206(10)62589-6 10071517

[27] Yonekura S, Okamoto Y, Horiguchi S, Sakurai D, Chazono H, Hanazawa T, et al Effects of aging on the natural history of seasonal allergic rhinitis in middle-aged subjects in South chiba, Japan. International archives of allergy and immunology. 2012;157(1):73–80. 10.1159/000324475 21912176

[28] Rondon C, Dona I, Torres MJ, Campo P, Blanca M. Evolution of patients with nonallergic rhinitis supports conversion to allergic rhinitis. The Journal of allergy and clinical immunology. 2009;123(5):1098–102. 10.1016/j.jaci.2009.02.018 19361848

[29] Rondon C, Canto G, Blanca M. Local allergic rhinitis: a new entity, characterization and further studies. Current opinion in allergy and clinical immunology. 2010;10(1):1–7. 10.1097/ACI.0b013e328334f5fb 20010094

[30] Mehra R, Redline S. Sleep apnea: a proinflammatory disorder that coaggregates with obesity. The Journal of allergy and clinical immunology. 2008;121(5):1096–102. 10.1016/j.jaci.2008.04.002 18466782 PMC2720266

[31] Wickwire EM, Collop NA. Insomnia and sleep-related breathing disorders. Chest. 2010;137(6):1449–63. 10.1378/chest.09-1485 20525657

[32] Gooneratne NS, Gehrman PR, Nkwuo JE, Bellamy SL, Schutte-Rodin S, Dinges DF, et al Consequences of comorbid insomnia symptoms and sleep-related breathing disorder in elderly subjects. Archives of internal medicine. 2006;166(16):1732–8. 10.1001/archinte.166.16.1732 16983051

[33] Krell SB, Kapur VK. Insomnia complaints in patients evaluated for obstructive sleep apnea. Sleep & breathing = Schlaf & Atmung. 2005;9(3):104–10. 16091954 10.1007/s11325-005-0026-x

[34] Ferguson BJ. Influences of allergic rhinitis on sleep. Otolaryngology—head and neck surgery: official journal of American Academy of Otolaryngology-Head and Neck Surgery. 2004;130(5):617–29. 15138430 10.1016/j.otohns.2004.02.001

[35] Corboz MR, Rivelli MA, Mingo GG, McLeod RL, Varty L, Jia Y, et al Mechanism of decongestant activity of alpha 2-adrenoceptor agonists. Pulmonary pharmacology & therapeutics. 2008;21(3):449–54. 17869148 10.1016/j.pupt.2007.06.007

[36] Pokladnikova J, Meyboom RH, Vlcek J, Edwards RI. Intranasally administered corticosteroids and neuropsychiatric disturbances: a review of the international pharmacovigilance programme of the World Health Organization. Annals of allergy, asthma & immunology: official publication of the American College of Allergy, Asthma, & Immunology. 2008;101(1):67–73. 10.1016/S1081-1206(10)60837-X 18681087

[37] Kim MY, Jo EJ, Kang SY, Chang YS, Yoon IY, Cho SH, et al Obstructive sleep apnea is associated with reduced quality of life in adult patients with asthma. Annals of allergy, asthma & immunology: official publication of the American College of Allergy, Asthma, & Immunology. 2013;110(4):253–7, 7 e1. 10.1016/j.anai.2013.01.005 23535088

[38] Teodorescu M, Polomis DA, Hall SV, Teodorescu MC, Gangnon RE, Peterson AG, et al Association of obstructive sleep apnea risk with asthma control in adults. Chest. 2010;138(3):543–50. 10.1378/chest.09-3066 20495105 PMC2940069

[39] Yigla M, Tov N, Solomonov A, Rubin AH, Harlev D. Difficult-to-control asthma and obstructive sleep apnea. The Journal of asthma: official journal of the Association for the Care of Asthma. 2003;40(8):865–71. 14736085 10.1081/jas-120023577

[40] Teodorescu M, Consens FB, Bria WF, Coffey MJ, McMorris MS, Weatherwax KJ, et al Predictors of habitual snoring and obstructive sleep apnea risk in patients with asthma. Chest. 2009;135(5):1125–32. 10.1378/chest.08-1273 18849401

[41] Larsson LG, Lindberg A, Franklin KA, Lundback B. Symptoms related to obstructive sleep apnoea are common in subjects with asthma, chronic bronchitis and rhinitis in a general population. Respiratory medicine. 2001;95(5):423–9. 10.1053/rmed.2001.1054 11392586

[42] Teodorescu M, Barnet JH, Hagen EW, Palta M, Young TB, Peppard PE. Association between asthma and risk of developing obstructive sleep apnea. Jama. 2015;313(2):156–64. 10.1001/jama.2014.17822 25585327 PMC4334115

[43] Gibson PG, McDonald VM, Marks GB. Asthma in older adults. Lancet. 2010;376(9743):803–13. 10.1016/S0140-6736(10)61087-2 20816547

