# Correction: Impact of self-reported symptoms of allergic rhinitis and asthma on sleep disordered breathing and sleep disturbances in the elderly with polysomnography study

**DOI:** 10.1371/journal.pone.0176425

**Published:** 2017-04-19

**Authors:** Sae-Hoon Kim, Ha-Kyeong Won, Sung-Do Moon, Byung-Keun Kim, Yoon-Seok Chang, Ki-Woong Kim, In-Young Yoon

One of the affiliations for the sixth and seventh authors is not indicated. Ki-Woong Kim and In-Young Yoon are both affiliated with: Department of Psychiatry, Seoul National University College of Medicine, Seoul, South Korea.
