# Supplementary material for: Impact of self-reported symptoms of allergic rhinitis and asthma on sleep disordered breathing and sleep disturbances in the elderly with polysomnography study
Source: PLoS One. 2017 Feb 28;12(2):e0173075. doi: 10.1371/journal.pone.0173075 (PMC5330513; doi:10.1371/journal.pone.0173075)
Supplement: S1 Table — (DOCX) [file pone.0173075.s001.docx]

S1 Table, Dataset of key variables of the study subjects

| Subject No. | Age | Gender | BMI  (Kg/m^2^) | Neck circum  (cm) | AHI | Sleep efficiency  (%) | Current wheezing | Ever diagnosis, asthma | Asthma treatment, past 12mo | Rhinitis symptoms, past 12mo | Ever-diagnosis, allergic rhinitis | Allergic rhinitis treatment, past 12mo |
| --- | --- | --- | --- | --- | --- | --- | --- | --- | --- | --- | --- | --- |
| 1 | 88 | M | 24.8 | 39 | 24.9 | 44.2 | No | No | No | No | No | No |
| 2 | 75 | M | 24.1 | 41 | 1.7 | 70.5 | No | No | No | Yes | No | No |
| 3 | 64 | F | 27.9 | 43 | 6.7 | 92.4 | No | No | No | Yes | No | No |
| 4 | 75 | F | 22.5 | 36 | 0.5 | 51.3 | No | No | No | Yes | No | No |
| 5 | 71 | M | 25.3 | 40 | 0.7 | 57.7 | Yes | Yes | Yes | No | No | No |
| 6 | 66 | M | 21.9 | 38 | 56.9 | 76.2 | No | No | No | Yes | Yes | Yes |
| 7 | 66 | F | 28.6 | 38.5 | 1.5 | 59.1 | No | No | No | No | No | No |
| 8 | 80 | F | 30.9 | 37 | 43.3 | 54.9 | No | Yes | No | Yes | No | No |
| 9 | 61 | M | 23.7 | 41 | 13.8 | 89 | No | No | No | Yes | No | No |
| 10 | 62 | M | 31.9 | 54 | 8.1 | 84.6 | No | No | No | No | No | No |
| 11 | 67 | F | 24.2 | 37 | 5.6 | 82.8 | No | No | No | No | No | No |
| 12 | 72 | M | 24.4 | 40 | 27.6 | 87.3 | No | No | No | No | No | No |
| 13 | 71 | M | 22.3 | 41 | 9.5 | 64.6 | No | No | No | Yes | No | No |
| 14 | 61 | M | 23.7 | 40 | 8.1 | 53.9 | Yes | No | No | *IR | IR | IR |
| 15 | 70 | M | 22.7 | 41 | 30 | 66.7 | No | Yes | Yes | Yes | Yes | No |
| 16 | 69 | M | 21.5 | 37 | 2.4 | 42.1 | No | Yes | No | Yes | Yes | No |
| 17 | 60 | M | 24.2 | 42 | 9.5 | 61.9 | No | No | No | Yes | No | No |
| 18 | 63 | F | 20.9 | 32.5 | 7.3 | 63.2 | No | No | No | Yes | No | No |
| 19 | 68 | F | 24.9 | 36 | 10.7 | 85.9 | No | No | No | No | No | No |
| 20 | 64 | F | 22.5 | 34 | 23.9 | 92.4 | No | No | No | Yes | Yes | No |
| 21 | 62 | F | 24.5 | 34 | 10.9 | 93.3 | No | No | No | No | No | No |
| 22 | 68 | F | 22.5 | 34 | 0.8 | 78.5 | No | No | No | No | No | No |
| 23 | 61 | F | 21.2 | 43 | 5.7 | 88 | No | No | No | Yes | Yes | No |
| 24 | 60 | F | 20.7 | 36 | 3.3 | 94.9 | No | No | No | Yes | No | No |
| 25 | 64 | F | 27.2 | 38 | 1.8 | 95.1 | No | No | No | Yes | No | No |
| 26 | 65 | F | 24 | 35 | 5.7 | 65.6 | No | No | No | No | No | No |
| 27 | 68 | M | 16.5 | 35 | 7.1 | 65.8 | No | No | No | No | No | No |
| 28 | 68 | F | 22.6 | 36 | 0 | 50.9 | No | No | No | No | No | No |
| 29 | 65 | F | 19.7 | 35 | 0 | 86.4 | No | No | No | No | No | No |
| 30 | 66 | F | 22.1 | 38 | 7.5 | 83.4 | No | No | No | No | No | No |
| 31 | 71 | M | 24.4 | 41 | 46.8 | 86.6 | No | No | No | No | No | No |
| 32 | 70 | M | 21.9 | 40 | 2.3 | 55.2 | No | No | No | Yes | No | No |
| 33 | 72 | M | 23.4 | 42 | 11.5 | 75.1 | No | No | No | Yes | Yes | No |
| 34 | 66 | F | 24 | 38 | 16.8 | 79.9 | No | No | No | No | No | No |
| 35 | 62 | M | 22 | 40 | 26 | 71.3 | No | No | No | No | No | No |
| 36 | 72 | F | 21 | 35 | 6.7 | 56.1 | No | No | No | No | No | No |
| 37 | 61 | F | 22.2 | 33 | 1.7 | 85.3 | No | No | No | No | No | No |
| 38 | 63 | F | 20.9 | 34 | 16.1 | 47.1 | No | No | No | Yes | No | No |
| 39 | 74 | F | 20.8 | 37 | 6.3 | 72.8 | No | No | No | Yes | No | No |
| 40 | 78 | M | 23.4 | 38 | 14.2 | 89.5 | No | No | No | No | No | No |
| 41 | 73 | F | 26 | 39 | 16.6 | 91.7 | No | No | No | No | No | No |
| 42 | 65 | F | 26.7 | 40 | 6.8 | 53.2 | No | No | No | No | No | No |
| 43 | 76 | M | 27.1 | 42 | 25.7 | 64.6 | No | No | No | No | No | No |
| 44 | 67 | M | 28.7 | 44 | 16.4 | 69.3 | Yes | No | No | Yes | No | No |
| 45 | 61 | F | 24.9 | 37 | 51.9 | 46.9 | No | Yes | Yes | Yes | Yes | Yes |
| 46 | 62 | F | 22.5 | 36 | 3.9 | 50.4 | Yes | No | No | Yes | Yes | Yes |
| 47 | 73 | M | 27.2 | 44 | 36.2 | 85.7 | Yes | No | No | Yes | No | No |
| 48 | 61 | F | 25.3 | 38 | 14.8 | 93 | No | No | No | No | No | No |
| 49 | 68 | F | 24.3 | 37 | 22.5 | 78.3 | No | No | No | No | Yes | No |
| 50 | 71 | M | 20.8 | 41 | 4.8 | 86.6 | No | No | No | Yes | No | No |
| 51 | 71 | F | 19.1 | 32 | 0.4 | 62.7 | No | No | No | No | No | No |
| 52 | 71 | F | 22.6 | 34 | 1.1 | 89.8 | No | No | No | No | No | No |
| 53 | 63 | M | 23.5 | 40 | 21.9 | 93.9 | No | No | No | No | IR | No |
| 54 | 76 | M | 29.8 | 47 | 50.4 | 42.6 | No | No | No | No | Yes | No |
| 55 | 60 | F | 21.3 | 33 | 0.7 | 70.3 | No | Yes | Yes | No | No | No |
| 56 | 85 | F | 19.2 | 35 | 7.6 | 71.4 | No | IR | No | No | No | No |
| 57 | 73 | F | 20.3 | 34 | 8.6 | 80.7 | No | No | No | Yes | No | No |
| 58 | 63 | F | 26.2 | 38 | 51.3 | 59.8 | No | No | No | No | No | No |
| 59 | 65 | F | 25.2 | 36.5 | 3.7 | 64.7 | No | No | No | No | No | No |
| 60 | 64 | F | 28.3 | 39 | 15.5 | 72.1 | No | IR | IR | Yes | Yes | No |
| 61 | 66 | F | 20.6 | 34 | 24.4 | 89.5 | No | No | No | No | No | No |
| 62 | 63 | F | 25 | 36 | 14.7 | 93.2 | No | No | No | Yes | No | No |
| 63 | 75 | M | 23.9 | 39 | 10.2 | 83.5 | No | No | No | No | No | No |
| 64 | 79 | M | 21 | 37 | 5.5 | 91.7 | No | No | No | No | No | No |
| 65 | 66 | M | 23.1 | 40 | 6.8 | 87.3 | No | No | No | No | No | No |
| 66 | 79 | F | 26 | 36 | 33 | 53 | No | No | No | Yes | Yes | Yes |
| 67 | 64 | M | 24.2 | 39 | 3.9 | 80.3 | No | No | No | No | No | No |
| 68 | 61 | F | 21.2 | 38 | 5.4 | 83.2 | No | No | No | No | No | No |
| 69 | 64 | M | 22.8 | 37 | 0.9 | 84.4 | No | No | No | No | No | No |
| 70 | 74 | F | 18 | 33 | 14.4 | 72.9 | No | No | No | No | No | No |
| 71 | 62 | F | 26.4 | 34 | 8.2 | 67.3 | No | No | No | No | Yes | No |
| 72 | 81 | M | 22.7 | 40 | 15.4 | 70.9 | No | No | No | No | No | No |
| 73 | 66 | F | 21.6 | 36 | 12.7 | 81.7 | Yes | No | No | No | No | No |
| 74 | 61 | F | 25.2 | 35 | 2.8 | 76 | Yes | Yes | No | No | No | No |
| 75 | 69 | M | 21.2 | 39 | 7.2 | 66.1 | No | No | No | No | No | No |
| 76 | 69 | F | 26 | 34 | 7.3 | 86 | No | No | No | No | No | No |
| 77 | 68 | M | 22.7 | 40 | 26.1 | 70.6 | No | No | No | No | No | No |
| 78 | 71 | M | 25 | 40 | 13.8 | 84.3 | No | No | No | Yes | No | No |
| 79 | 71 | F | 23 | 30 | 7.8 | 78.3 | No | No | No | No | No | No |
| 80 | 79 | M | 24.5 | 40 | 41.3 | 85.4 | No | No | No | Yes | No | No |
| 81 | 64 | F | 22.2 | 35 | 6.7 | 91.4 | No | No | No | No | No | No |
| 82 | 65 | F | 20.5 | 33.5 | 7.3 | 88.8 | No | No | No | No | No | No |
| 83 | 77 | M | 25.4 | 41 | 42.6 | 88.9 | No | No | No | Yes | Yes | Yes |
| 84 | 72 | F | 25.5 | 34 | 32.2 | 83.6 | No | No | No | Yes | No | No |
| 85 | 62 | F | 21.1 | 39 | 3.5 | 82 | No | No | No | Yes | No | No |
| 86 | 76 | M | 23.6 | 38 | 34.3 | 46.1 | No | No | No | Yes | No | No |
| 87 | 71 | F | 21.5 | 35 | 6.2 | 76.8 | No | No | No | No | No | No |
| 88 | 61 | M | 24.9 | 40 | 22.6 | 81.3 | No | No | No | No | No | No |
| 89 | 69 | M | 23.1 | 39 | 8.3 | 87.1 | No | No | No | No | No | No |
| 90 | 84 | F | 23.8 | 33 | 13.9 | 66.8 | No | No | No | No | No | No |
| 91 | 75 | F | 23.5 | 33 | 13.7 | 71.2 | No | No | No | No | Yes | IR |
| 92 | 66 | F | 21.9 | 36 | 3.8 | 75.5 | No | No | No | Yes | Yes | Yes |
| 93 | 75 | F | 25.2 | 37 | 39.3 | 82.6 | No | No | No | No | No | No |
| 94 | 61 | F | 20.4 | 34.5 | 1.6 | 74.1 | No | No | No | IR | Yes | Yes |
| 95 | 73 | F | 26.8 | 39 | 8.7 | 92 | No | No | No | No | No | No |
| 96 | 62 | F | 21.8 | 34 | 0.1 | 94.2 | No | No | No | No | No | No |
| 97 | 68 | F | 20.6 | 36 | 7.2 | 46.9 | Yes | No | No | Yes | Yes | Yes |
| 98 | 61 | F | 26.7 | 35 | 3.4 | 94.9 | No | No | No | Yes | Yes | Yes |
| 99 | 68 | F | 22.3 | 37 | 9 | 61.1 | No | No | No | Yes | No | No |
| 100 | 62 | M | 24.2 | 40 | 41.7 | 96 | No | No | No | No | No | No |
| 101 | 69 | M | 20 | 37 | 83.5 | 49.8 | No | Yes | Yes | No | No | No |
| 102 | 67 | F | 24.1 | 37 | 5.4 | 43.2 | No | No | No | No | No | No |
| 103 | 65 | F | 27 | 40 | 31.3 | 94.5 | No | No | No | No | No | No |
| 104 | 64 | M | 20.1 | 36 | 25.3 | 88.9 | No | No | No | No | No | No |
| 105 | 65 | M | 29.4 | 45 | 31.6 | 81.5 | No | No | No | No | No | No |
| 106 | 64 | M | 24.6 | 38.5 | 3.3 | 83.2 | No | No | No | No | No | No |
| 107 | 65 | F | 22.8 | 35 | 1.3 | 77.7 | No | No | No | No | No | No |
| 108 | 76 | M | 21.4 | 40 | 5.4 | 94.4 | No | No | No | No | No | No |
| 109 | 81 | M | 31.7 | 42 | 47.3 | 65.6 | No | No | No | No | No | No |
| 110 | 77 | F | 23.4 | 37.5 | 18.9 | 79.2 | No | No | No | No | Yes | Yes |
| 111 | 66 | M | 24.3 | 41 | 12.8 | 73.8 | No | No | No | Yes | No | No |
| 112 | 73 | M | 24.2 | 42 | 29.4 | 81.1 | No | No | No | Yes | No | No |
| 113 | 63 | F | 21.4 | 34 | 43.4 | 76.1 | No | No | No | No | No | No |
| 114 | 66 | F | 23.2 | 35 | 5.9 | 80.6 | IR | IR | IR | IR | IR | IR |
| 115 | 84 | M | 27.2 | 38.5 | 56.1 | 70.3 | No | No | No | No | No | No |
| 116 | 70 | F | 22.9 | 34.5 | 8.2 | 73 | No | No | No | No | No | No |
| 117 | 75 | F | 24.8 | 36.5 | 18 | 65.5 | No | No | No | No | No | No |
| 118 | 60 | F | 26 | 37 | 7.3 | 81.7 | No | No | No | No | No | No |
| 119 | 78 | F | 20.4 | 33 | 13.6 | 71.7 | No | No | No | No | No | No |
| 120 | 63 | F | 24.7 | 35 | 8.1 | 85.4 | No | No | No | Yes | Yes | No |
| 121 | 68 | F | 27.9 | 39 | 15.9 | 94.6 | Yes | No | No | No | Yes | Yes |
| 122 | 69 | M | 22.2 | 41 | 16.3 | 85.4 | No | No | No | IR | IR | IR |
| 123 | 76 | M | 21.3 | 36 | 13.5 | 75.7 | No | No | No | No | No | No |
| 124 | 64 | F | 27.9 | 38 | 1.5 | 83.7 | No | No | No | No | No | No |
| 125 | 63 | M | 20 | 39 | 3.1 | 72.5 | No | No | No | Yes | No | No |
| 126 | 69 | F | 21.5 | 34 | 2.7 | 82.1 | No | No | No | No | No | No |
| 127 | 61 | F | 27.9 | 37 | 24.8 | 85 | No | No | No | Yes | Yes | No |
| 128 | 62 | F | 22.5 | 34 | 2.6 | 90.3 | No | No | No | No | No | No |
| 129 | 68 | F | 22.5 | 35 | 0.8 | 69.5 | No | No | No | No | No | No |
| 130 | 68 | M | 22.5 | 39 | 8.5 | 95.8 | No | No | No | Yes | No | No |
| 131 | 63 | M | 22.8 | 40.5 | 59.1 | 75.5 | No | No | No | No | No | No |
| 132 | 60 | F | 24 | 35.5 | 3 | 88.3 | No | No | No | No | No | No |
| 133 | 62 | M | 23.5 | 42 | 1.5 | 80.8 | No | No | No | No | No | No |
| 134 | 67 | F | 26.3 | 37 | 5.9 | 86.7 | No | No | No | No | No | No |
| 135 | 66 | F | 24.3 | 37 | 14 | 88.3 | No | No | No | No | No | No |
| 136 | 66 | F | 21.4 | 33 | 0.8 | 73.8 | Yes | Yes | Yes | Yes | Yes | Yes |
| 137 | 77 | M | 24 | 38 | 9 | 76.6 | No | No | No | No | No | No |
| 138 | 69 | F | 26.2 | 37 | 9.2 | 87.4 | No | No | No | IR | No | No |
| 139 | 69 | F | 26.1 | 38 | 7.5 | 84.5 | No | No | No | No | No | No |
| 140 | 68 | F | 18.6 | 34 | 2.7 | 53.9 | No | No | No | No | No | No |
| 141 | 77 | F | 26.2 | 40 | 3.2 | 47.4 | No | No | No | Yes | No | No |
| 142 | 68 | F | 21.2 | 35 | 4.7 | 91.1 | No | No | No | No | No | No |
| 143 | 69 | F | 23.1 | 34 | 14.4 | 86.2 | No | No | No | Yes | Yes | No |
| 144 | 65 | F | 19.4 | 32 | 10 | 87.3 | No | No | No | Yes | No | No |
| 145 | 68 | M | 22.5 | 40 | 4.7 | 72.7 | No | No | No | No | No | No |
| 146 | 64 | F | 18.1 | 33 | 20.2 | 92.2 | No | No | No | No | No | No |
| 147 | 67 | F | 24.2 | 36 | 3.9 | 67.7 | Yes | Yes | Yes | No | Yes | Yes |
| 148 | 79 | F | 25 | 37 | 12.4 | 74.5 | Yes | No | No | No | No | No |
| 149 | 60 | F | 27.8 | 35 | 0.9 | 79 | Yes | No | No | Yes | Yes | No |
| 150 | 66 | M | 21.6 | 40 | 8.6 | 64.1 | No | No | No | Yes | Yes | Yes |
| 151 | 60 | M | 25.7 | 44 | 38.3 | 77.5 | No | No | No | No | No | No |
| 152 | 70 | F | 22.5 | 38 | 7.4 | 86.3 | No | No | No | No | No | No |
| 153 | 72 | F | 24.7 | 37 | 3.2 | 78 | No | No | No | No | No | No |
| 154 | 67 | F | 25.7 | 32 | 0.8 | 77.5 | No | No | No | No | No | No |
| 155 | 73 | M | 23.4 | 39 | 6.7 | 58.6 | No | No | No | Yes | No | No |
| 156 | 70 | M | 23.3 | 41 | 4.7 | 79.5 | No | No | No | IR | No | No |
| 157 | 72 | F | 24.7 | 38 | 5.4 | 71.7 | No | No | No | No | No | No |
| 158 | 66 | F | 21.3 | 35 | 1.9 | 93.2 | No | No | No | No | No | No |
| 159 | 70 | F | 23.4 | 33 | 4.4 | 52.6 | No | No | No | No | No | No |
| 160 | 61 | M | 25.5 | 39 | 5.4 | 86.2 | No | No | No | No | No | No |
| 161 | 69 | F | 26.4 | 36 | 14.1 | 78.3 | Yes | Yes | Yes | No | No | No |
| 162 | 78 | M | 23.9 | 37 | 65.9 | 74.6 | No | No | No | Yes | No | No |
| 163 | 63 | M | 28.4 | 42 | 18.2 | 72.3 | No | No | No | No | No | No |
| 164 | 69 | M | 21.3 | 38 | 42.2 | 61 | No | No | No | No | No | No |
| 165 | 71 | F | 18.8 | 35 | 39.8 | 67.3 | No | No | No | No | No | No |
| 166 | 69 | M | 19.9 | 36 | 33.1 | 75.9 | No | No | No | No | No | No |
| 167 | 63 | F | 22 | 33 | 2.6 | 94.3 | No | Yes | No | No | No | No |
| 168 | 68 | M | 22.2 | 37 | 35.9 | 76.4 | No | No | No | Yes | No | No |
| 169 | 73 | F | 23.1 | 35 | 1.8 | 63.8 | No | No | No | No | No | No |
| 170 | 63 | F | 22.6 | 34 | 17.2 | 88 | No | No | No | No | No | No |
| 171 | 64 | F | 21.4 | 33 | 3.6 | 95.2 | No | No | No | No | No | No |
| 172 | 75 | M | 26.8 | 45 | 57.8 | 51.4 | No | No | No | No | No | No |
| 173 | 69 | F | 26.1 | 36 | 7.2 | 84.9 | No | No | No | Yes | Yes | No |
| 174 | 60 | F | 23.6 | 34 | 0 | 54.1 | No | No | No | No | No | No |
| 175 | 68 | F | 26 | 37 | 11.5 | 93.1 | No | No | No | No | No | No |
| 176 | 74 | F | 22.8 | 35 | 1.7 | 86.3 | Yes | No | No | No | No | No |
| 177 | 71 | F | 23.5 | 35.5 | 4.1 | 80.2 | Yes | Yes | Yes | No | No | No |
| 178 | 74 | F | 31.2 | 38 | 20.3 | 38 | Yes | Yes | Yes | No | No | No |
| 179 | 68 | F | 22.8 | 40 | 25.7 | 79.8 | No | No | No | No | Yes | Yes |
| 180 | 73 | M | 25.7 | 42.5 | 22.2 | 82.1 | No | No | No | Yes | Yes | Yes |
| 181 | 65 | F | 26 | 35 | 0.5 | 72 | No | No | No | No | No | No |
| 182 | 68 | F | 23.2 | 34 | 0 | 42.4 | No | No | No | No | No | No |
| 183 | 72 | F | 27.4 | 37 | 3.4 | 90.1 | Yes | No | No | No | No | No |
| 184 | 61 | F | 23.1 | 38 | 21.3 | 91.3 | No | No | No | No | No | No |
| 185 | 60 | F | 22.6 | 35 | 0.3 | 91.2 | No | No | No | Yes | No | No |
| 186 | 88 | F | 23.9 | 35 | 5.8 | 74.8 | No | No | No | No | No | No |
| 187 | 73 | F | 24 | 36 | 11.9 | 92.6 | No | No | No | No | No | No |
| 188 | 77 | F | 20.5 | 33 | 3.1 | 53.9 | No | No | No | No | No | No |
| 189 | 62 | F | 25.4 | 34 | 15.7 | 87.7 | No | No | No | No | Yes | No |
| 190 | 69 | M | 23.2 | 37 | 29.6 | 89 | No | No | No | Yes | Yes | No |
| 191 | 69 | M | 19.4 | 34 | 10.9 | 71.4 | No | No | No | No | No | No |
| 192 | 61 | M | 24.1 | 38 | 9.4 | 93.9 | No | No | No | No | No | No |
| 193 | 69 | M | 23.8 | 39.5 | 6.6 | 81.9 | Yes | No | No | No | No | No |
| 194 | 68 | F | 22.2 | 35 | 10.6 | 73.9 | No | No | No | No | No | No |
| 195 | 61 | M | 26.7 | 43 | 60.5 | 96.5 | No | No | No | No | No | No |
| 196 | 70 | F | 22.5 | 35 | 5 | 72.6 | No | No | No | Yes | No | No |
| 197 | 64 | F | 23.2 | 37 | 17.5 | 86 | No | IR | IR | No | No | No |
| 198 | 84 | F | 20.5 | 36 | 5.9 | 60.1 | No | Yes | Yes | No | No | No |
| 199 | 62 | M | 23.4 | 38 | 42.8 | 90.8 | No | No | No | No | No | No |
| 200 | 67 | M | 23.2 | 41.5 | 15.8 | 66.6 | No | No | No | Yes | Yes | Yes |
| 201 | 64 | F | 21.7 | 39 | 9 | 91.1 | No | No | No | No | No | No |
| 202 | 61 | F | 22.2 | 34 | 3 | 86 | No | No | No | Yes | No | No |
| 203 | 75 | M | 25.4 | 42 | 48.2 | 84.8 | No | No | No | No | No | No |
| 204 | 68 | M | 32.3 | 56 | 20.2 | 67.5 | No | No | No | Yes | Yes | Yes |
| 205 | 71 | M | 27.1 | 43 | 44.6 | 73.4 | No | No | No | No | No | No |
| 206 | 65 | F | 22.7 | 35 | 1.8 | 70.6 | No | No | No | No | No | No |
| 207 | 60 | M | 25.3 | 41 | 53.4 | 73.7 | No | No | No | Yes | No | No |
| 208 | 78 | M | 18.6 | 33 | 4 | 38.1 | No | No | No | Yes | Yes | Yes |
| 209 | 70 | F | 26.4 | 37 | 46.4 | 60.8 | No | No | No | No | No | No |
| 210 | 63 | F | 24.3 | 35 | 0.6 | 94.9 | No | IR | No | No | IR | No |
| 211 | 70 | M | 22.2 | 38 | 37.6 | 76.7 | No | No | No | No | No | No |
| 212 | 70 | F | 26 | 35 | 10 | 93 | No | No | No | Yes | No | No |
| 213 | 64 | M | 24.2 | 39 | 13.3 | 83 | No | Yes | No | No | No | No |
| 214 | 72 | F | 19.1 | 32.5 | 0.2 | 72.9 | IR | No | No | No | No | No |
| 215 | 69 | F | 21.5 | 38 | 32.1 | 55.4 | No | No | No | No | No | No |
| 216 | 61 | F | 27.2 | 38 | 4.4 | 91.5 | No | No | No | No | No | No |
| 217 | 62 | F | 31.2 | 41 | 6.3 | 78.3 | No | No | No | No | No | No |
| 218 | 60 | M | 22.8 | 43 | 17.6 | 93.1 | No | No | No | No | No | No |
| 219 | 69 | M | 27.5 | 41.5 | 21.6 | 86 | No | No | No | No | No | No |
| 220 | 69 | M | 22.6 | 38 | 35.4 | 84.9 | No | No | No | No | No | No |
| 221 | 63 | M | 23.1 | 39.5 | 3.2 | 84 | No | No | No | No | No | No |
| 222 | 66 | M | 24.8 | 44 | 30.4 | 94.7 | No | No | No | No | No | No |
| 223 | 72 | F | 26.6 | 34 | 36.2 | 81.9 | No | No | No | Yes | No | No |
| 224 | 61 | F | 20.8 | 38 | 6 | 55.9 | No | No | No | No | No | No |
| 225 | 73 | F | 26.7 | 12 | 8.4 | 88.9 | No | No | No | IR | IR | IR |
| 226 | 67 | F | 25.9 | 38 | 9 | 60.7 | No | No | No | No | No | No |
| 227 | 65 | F | 23 | 39 | 29 | 92 | No | Yes | Yes | Yes | No | No |
| 228 | 74 | F | 24.4 | 35 | 5.7 | 59.1 | No | No | No | No | No | No |
| 229 | 71 | F | 23.2 | 38 | 1.1 | 77.1 | No | No | No | IR | No | No |
| 230 | 72 | F | 26.1 | 36.5 | 18.4 | 84.5 | No | No | No | No | No | No |
| 231 | 64 | F | 23 | 36 | 25.5 | 51.5 | No | No | No | No | No | No |
| 232 | 63 | F | 26.1 | 39 | 7.7 | 71.2 | No | IR | IR | IR | IR | IR |
| 233 | 62 | M | 18.3 | 42 | 2.3 | 87 | No | No | No | No | No | No |
| 234 | 71 | F | 25.3 | 36 | 19.8 | 61.6 | No | No | No | No | No | No |
| 235 | 70 | M | 21.5 | 39 | 12.6 | 93.6 | IR | IR | IR | IR | IR | IR |
| 236 | 74 | M | 24.8 | 43 | 8.7 | 85.6 | No | No | No | Yes | No | No |
| 237 | 70 | F | 23.2 | 38 | 6 | 95.4 | No | No | No | No | Yes | No |
| 238 | 64 | F | 24.5 | 38 | 10.4 | 81.2 | No | No | No | No | No | No |
| 239 | 64 | F | 23 | 34 | 3.3 | 47.2 | No | No | No | Yes | No | No |
| 240 | 66 | M | 29.1 | 43 | 24.5 | 80.7 | No | No | No | Yes | Yes | No |
| 241 | 65 | F | 23.3 | 42 | 3.3 | 90.2 | No | No | No | No | No | No |
| 242 | 71 | M | 21.1 | 42 | 23 | 78.7 | No | No | No | Yes | Yes | Yes |
| 243 | 64 | M | 22.9 | 39 | 7 | 87.1 | No | No | No | Yes | Yes | Yes |
| 244 | 69 | M | 23 | 40 | 3.3 | 83.8 | No | No | No | Yes | Yes | Yes |
| 245 | 64 | F | 22.2 | 40 | 14.7 | 85.3 | No | No | No | No | No | No |
| 246 | 65 | F | 23.3 | 37 | 17.6 | 67.4 | No | Yes | Yes | Yes | Yes | Yes |
| 247 | 67 | F | 22.4 | 37.5 | 7.4 | 87.6 | No | No | No | No | No | No |
| 248 | 68 | F | 22.8 | 33 | 2 | 63.4 | No | No | No | No | No | No |
| 249 | 68 | M | 25.2 | 41 | 27.2 | 53.2 | No | No | No | Yes | Yes | Yes |
| 250 | 63 | F | 23.3 | 39 | 15.6 | 87.4 | No | No | No | No | No | No |
| 251 | 72 | M | 23.1 | 39 | 9.5 | 62.9 | Yes | No | No | No | No | No |
| 252 | 66 | F | 25.7 | 37 | 11.2 | 81.1 | No | No | No | No | No | No |
| 253 | 62 | M | 20.9 | 36 | 1.6 | 66 | No | No | No | No | No | No |
| 254 | 71 | M | 24.5 | 43.5 | 4.9 | 83.5 | Yes | No | No | No | No | No |
| 255 | 67 | F | 24.9 | 34 | 9.6 | 83.1 | No | No | No | Yes | No | No |
| 256 | 77 | F | 24.8 | 32 | 0.8 | 88.9 | No | IR | IR | IR | IR | IR |
| 257 | 64 | F | 20.3 | 37 | 16.3 | 81.9 | No | No | No | No | No | No |
| 258 | 73 | F | 19.4 | 40 | 18.7 | 80.3 | No | No | No | Yes | No | No |
| 259 | 66 | F | 26.8 | 37.5 | 27.1 | 82.4 | No | No | No | No | No | No |
| 260 | 72 | F | 24.6 | 37.5 | 3.4 | 68.3 | No | No | No | No | No | No |
| 261 | 76 | M | 19.5 | 39 | 2.2 | 89.4 | No | No | No | Yes | No | No |
| 262 | 68 | M | 25.1 | 38 | 36 | 92.1 | No | No | No | No | No | No |
| 263 | 67 | M | 22.1 | 38 | 21.3 | 80.7 | No | No | No | No | No | No |
| 264 | 65 | M | 21.7 | 8 | 48.9 | 61.3 | No | No | No | Yes | Yes | Yes |
| 265 | 71 | F | 21.8 | 33 | 27.7 | 55.5 | No | No | No | No | No | No |
| 266 | 76 | F | 21 | 35 | 1.9 | 78.2 | No | No | No | No | No | No |
| 267 | 76 | M | 20 | 34 | 4.8 | 77.5 | No | No | No | Yes | No | No |
| 268 | 64 | F | 25 | 35 | 11.1 | 90.9 | No | No | No | Yes | Yes | No |
| 269 | 80 | F | 26.2 | 37 | 11.8 | 70.7 | No | No | No | No | No | No |
| 270 | 61 | M | 23.4 | 36 | 1.2 | 75.2 | No | No | No | No | No | No |
| 271 | 66 | F | 21 | 33 | 17.4 | 64.3 | No | No | No | No | No | No |
| 272 | 70 | M | 19.7 | 38 | 21.2 | 40.3 | No | No | No | No | No | No |
| 273 | 72 | M | 24.5 | 43 | 36.1 | 88.1 | No | No | No | No | No | No |
| 274 | 78 | F | 30.4 | 39 | 44.2 | 93.2 | No | No | No | No | No | No |
| 275 | 65 | F | 27.9 | 35 | 0.7 | 86.9 | No | No | No | No | No | No |
| 276 | 73 | M | 19.6 | 38 | 9 | 88 | Yes | Yes | Yes | No | No | No |
| 277 | 70 | F | 23.2 | 35 | 27.7 | 73.4 | No | No | No | No | No | No |
| 278 | 73 | F | 25.8 | 34 | 3.3 | 92.6 | No | Yes | No | Yes | Yes | No |
| 279 | 76 | F | 23.1 | 33.5 | 3.1 | 86.6 | No | No | No | No | IR | IR |
| 280 | 67 | M | 20.2 | 37 | 3.2 | 91.3 | No | No | No | No | No | No |
| 281 | 80 | M | 19.8 | 38 | 20.5 | 81 | No | No | No | No | No | No |
| 282 | 66 | F | 27.9 | 36 | 4.1 | 85.4 | No | No | No | No | No | No |
| 283 | 79 | F | 22.6 | 36 | 6.1 | 76.6 | No | No | No | No | No | No |
| 284 | 65 | F | 28.6 | 36 | 23.5 | 90.6 | No | No | No | No | No | No |
| 285 | 66 | M | 26.7 | 42 | 20.8 | 84.6 | No | No | No | Yes | No | No |
| 286 | 72 | F | 25.2 | 37 | 9 | 95.9 | Yes | Yes | Yes | No | No | No |
| 287 | 67 | M | 25 | 37 | 22.8 | 86.7 | No | No | No | Yes | No | No |
| 288 | 64 | F | 21.7 | 36.5 | 0 | 86.9 | No | No | No | No | No | No |
| 289 | 66 | M | 27.8 | 42 | 15.9 | 70.5 | No | No | No | No | No | No |
| 290 | 66 | F | 19.9 | 34 | 1.1 | 91.4 | No | No | No | No | No | No |
| 291 | 62 | F | 26.2 | 38 | 9.1 | 74 | No | No | No | Yes | No | No |
| 292 | 67 | F | 23.1 | 38 | 12.3 | 95 | No | No | No | Yes | No | No |
| 293 | 65 | M | 23.6 | 43 | 9.7 | 87.1 | No | No | No | Yes | No | No |
| 294 | 69 | F | 25.1 | 37 | 13.8 | 79.4 | No | No | No | No | No | No |
| 295 | 70 | M | 24.3 | 38 | 24.9 | 89.1 | No | No | No | Yes | Yes | Yes |
| 296 | 67 | M | 24.8 | 39 | 31.7 | 60.8 | No | No | No | Yes | No | No |
| 297 | 66 | F | 25.9 | 38 | 40.8 | 79.9 | No | No | No | No | No | No |
| 298 | 65 | M | 26.7 | 41 | 26 | 77.4 | No | No | No | Yes | Yes | No |
| 299 | 69 | F | 21.2 | 35 | 2.3 | 89.7 | No | No | No | Yes | Yes | Yes |
| 300 | 71 | F | 24.8 | 33 | 1 | 86.7 | No | No | No | No | No | No |
| 301 | 65 | M | 28 | 43.5 | 23.6 | 89.6 | No | No | No | No | No | No |
| 302 | 64 | M | 25.5 | 40 | 34.9 | 82.3 | No | No | No | No | No | No |
| 303 | 63 | F | 27.5 | 36 | 8.9 | 76.3 | No | No | No | No | No | No |
| 304 | 68 | F | 17.5 | 33 | 33.3 | 88.3 | No | No | No | No | No | No |
| 305 | 62 | M | 24.1 | 43.5 | 22.6 | 79.5 | No | No | No | No | Yes | No |
| 306 | 64 | F | 20.4 | 35 | 11.4 | 82.1 | No | No | No | Yes | No | No |
| 307 | 61 | M | 19.5 | 38.5 | 3.1 | 89.9 | No | No | No | No | No | No |
| 308 | 75 | M | 22.3 | 37.5 | 6 | 76.5 | No | No | No | No | No | No |
| 309 | 75 | F | 29.8 | 37.5 | 4.5 | 78.2 | No | No | No | No | No | No |
| 310 | 68 | M | 24.5 | 39 | 39.8 | 82 | No | No | No | No | No | No |
| 311 | 62 | M | 23.3 | 43 | 0.5 | 65.5 | No | No | No | No | No | No |
| 312 | 72 | M | 20 | 36 | 49.1 | 66.6 | No | No | No | No | No | No |
| 313 | 78 | F | 19.6 | 31 | 19.3 | 75.7 | No | No | No | No | Yes | Yes |
| 314 | 68 | F | 24.1 | 36 | 25.8 | 84.8 | No | No | No | No | No | No |
| 315 | 65 | F | 31.4 | 39 | 27.2 | 89.5 | No | No | No | No | No | No |
| 316 | 78 | F | 26.9 | 37 | 17.4 | 74.1 | No | No | No | No | No | No |
| 317 | 62 | M | 19.7 | 35 | 8.9 | 81 | No | No | No | Yes | Yes | Yes |
| 318 | 67 | M | 27.1 | 43 | 19 | 78.3 | No | No | No | Yes | No | No |
| 319 | 65 | F | 25.8 | 38.5 | 1.3 | 74.8 | No | No | No | No | Yes | Yes |
| 320 | 70 | M | 25.2 | 51 | 23 | 45.6 | No | No | No | No | No | No |
| 321 | 74 | M | 24.6 | 42.5 | 14 | 60 | No | No | No | No | No | No |
| 322 | 75 | F | 21.8 | 38 | 5.5 | 78.9 | No | No | No | No | No | No |
| 323 | 66 | M | 23.7 | 42 | 10.2 | 68.5 | No | No | No | No | No | No |
| 324 | 66 | F | 26.8 | 37 | 44.8 | 83.6 | No | No | No | IR | No | No |
| 325 | 69 | F | 21.1 | 33.5 | 13.4 | 78.9 | No | No | No | No | No | No |
| 326 | 70 | M | 26.2 | 41.5 | 9.8 | 69.6 | No | No | No | Yes | Yes | Yes |
| 327 | 62 | F | 22.5 | 37 | 24.6 | 73.1 | No | IR | No | Yes | IR | No |
| 328 | 71 | M | 26.4 | 39 | 17.2 | 76.5 | No | No | No | No | No | No |
| 329 | 68 | F | 25.9 | 36 | 21.6 | 61.4 | No | No | No | No | No | No |
| 330 | 64 | F | 21.4 | 34 | 11.4 | 87.5 | No | No | No | No | No | No |
| 331 | 68 | M | 25.5 | 42 | 20.7 | 93.5 | No | No | No | No | Yes | No |
| 332 | 61 | M | 22.3 | 39.5 | 11.3 | 75.6 | No | No | No | No | Yes | No |
| 333 | 65 | F | 19.3 | 33.5 | 2.3 | 59.3 | No | No | No | No | No | No |
| 334 | 83 | F | 21.2 | 38.5 | 5.2 | 76 | No | No | No | No | No | No |
| 335 | 72 | F | 26 | 37.5 | 38 | 72 | No | No | No | No | No | No |
| 336 | 68 | F | 23.2 | 35.5 | 2.5 | 76.3 | No | No | No | No | No | No |
| 337 | 70 | F | 16.7 | 33 | 10.4 | 62.2 | No | No | No | No | Yes | Yes |
| 338 | 63 | F | 20.7 | 34 | 2.2 | 78.3 | No | No | No | No | No | No |
| 339 | 70 | F | 23.3 | 34 | 8.5 | 77.2 | No | No | No | No | No | No |
| 340 | 71 | F | 27 | 45 | 47.9 | 88.2 | No | No | No | No | No | No |
| 341 | 65 | F | 21 | 33 | 8.6 | 63.3 | No | No | No | No | No | No |
| 342 | 64 | F | 24.3 | 33 | 9.2 | 93.9 | No | No | No | No | No | No |
| 343 | 79 | M | 22.6 | 39.5 | 2.1 | 77.8 | No | No | No | IR | No | No |
| 344 | 66 | F | 21.9 | 32.5 | 1.1 | 52.9 | No | No | No | Yes | No | No |
| 345 | 79 | F | 24 | 37.5 | 48.1 | 81.3 | No | No | No | No | No | No |
| 346 | 60 | M | 22 | 38.5 | 7.7 | 46.1 | No | No | No | No | No | No |
| 347 | 60 | F | 28 | 38.5 | 5.7 | 76.9 | No | No | No | No | No | No |
| 348 | 69 | F | 26.6 | 36.5 | 3 | 66.5 | No | No | No | No | No | No |

*IR: invalid or incomplete response
